# Roxadustat for ESA‐Hyporesponsive Renal Anemia in a 13‐Year‐Old on Hemodialysis: Case Report and Mini‐Review

**DOI:** 10.1002/ccr3.70985

**Published:** 2025-09-26

**Authors:** Meng Zhang, Yanchao Guo, Yan Zhu, Juan Yang

**Affiliations:** ^1^ Department of Nephrology, Tongji Hospital, Tongji Medical College Huazhong University of Science and Technology Wuhan Hubei China; ^2^ Department of Clinical Nutrition, Tongji Hospital, Tongji Medical College Huazhong University of Science and Technology Wuhan Hubei China; ^3^ Blood Purification Center, Tongji Hospital, Tongji Medical College Huazhong University of Science and Technology Wuhan Hubei China

**Keywords:** adolescent, anemia, prolyl‐hydroxylase inhibitors, renal dialysis, Roxadustat

## Abstract

Anemia is the most common complication of end‐stage renal disease (ESRD). While erythropoiesis‐stimulating agents (ESA) have improved treatment outcomes, some patients exhibit intolerance or hyporesponsiveness. Roxadustat, a hypoxia‐inducible factor prolyl hydroxylase inhibitor (HIF‐PHI), improves erythropoiesis and iron metabolism, offering advantages in ESA‐refractory cases. However, its use in pediatric populations remains limited. We present a case of a 13‐year‐old girl on maintenance hemodialysis who exhibited both hyporesponsiveness and intolerance to ESA. Initiation of oral Roxadustat resulted in a sustained improvement in hemoglobin levels, accompanied by notable enhancements in iron metabolism and clinical symptoms. So far, over 5 years of follow‐up, no significant adverse events were observed. Oral Roxadustat appears to be an effective and safe treatment option for teenage hemodialysis patients who are resistant or intolerant to ESA.

## Graphical abstract

Changes in hemoglobin and iron metabolism parameters during Roxadustat treatment in a 13‐year‐old dialysis patient.

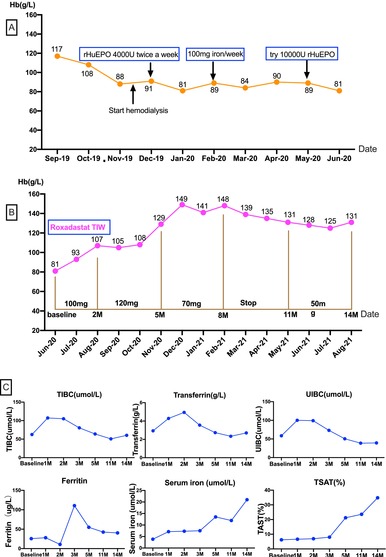

**Summary**

- In an adolescent on maintenance hemodialysis with ESA hyporesponsiveness and intolerance, oral Roxadustat achieved durable hemoglobin control, improved iron indices, and symptom relief without major adverse events over 5 years, suggesting a practical alternative when ESA therapy is ineffective or poorly tolerated in adolescents.

## 1 Introduction

Renal anemia is a common complication of chronic kidney disease (CKD), affecting up to 80% of dialysis patients [1], primarily due to erythropoietin (EPO) deficiency, with contributions from blood loss, iron imbalance, and inflammation [2]. Although erythropoiesis‐stimulating agents (ESA) therapy improves quality of life, some patients remain unresponsive [3], and ESAs carry risks such as hypertension, thrombosis, and increased mortality [3]. These limitations underscore the need for safer, more effective alternatives.

Roxadustat is an oral hypoxia‐inducible factor prolyl hydroxylase inhibitor (HIF‐PHI) that stabilizes HIF, increases endogenous EPO, and improves iron availability by lowering hepcidin [4]. Roxadustat is approved for CKD‐related anemia in both dialysis and non‐dialysis patients and has shown efficacy in ESA‐hyporesponsive adults on hemodialysis [5]. However, pediatric use remains off‐label in most regions, and high‐quality data are scarce, limited to only a handful of case reports/series and early pediatric trials that are limited and ongoing. Here, we report the successful use of Roxadustat in a 13‐year‐old hemodialysis patient with ESA hyporesponsiveness and intolerance, with 5‐year follow‐up.

## 2 Case Presentation

The patient was a 13‐year‐old girl presenting with recurrent episodes of dizziness, fatigue, and shortness of breath on exertion. She had resumed maintenance hemodialysis 1 month prior. Her medical history was significant for IgA nephropathy, diagnosed by renal biopsy in 2014. She progressed rapidly to end‐stage renal disease (ESRD) and initiated thrice‐weekly hemodialysis at the age of 9 in 2015. A living‐related renal transplant was performed in 2017, but the allograft failed in 2019 due to chronic rejection, necessitating the re‐initiation of maintenance hemodialysis.

On physical examination, the patient appeared pale but was hemodynamically stable, with a body temperature of 36.5°C, blood pressure of 150/100 mmHg, pulse rate of 90 beats per minute, and respiratory rate of 22 breaths per minute. No peripheral edema or signs of fluid overload were observed. Cardiac and pulmonary auscultation were unremarkable. Neurological and abdominal examinations were within normal limits.

Laboratory investigations demonstrated a reduced white blood cell count of 3.58 × 10^9^/L (normal range: 3.8–5.1 × 10^9^/L) and a hemoglobin level of 91 g/L (normal range: 115–150 g/L). Kidney function testing indicated a serum creatinine (Scr) concentration of 811 μmol/L (normal range: 45–84 μmol/L) and a severely decreased glomerular filtration rate (GFR) of 6 mL/min/1.73 m^2^ (normal range: 90–110 mL/min). Iron metabolism parameters revealed a serum ferritin (SF) concentration of 112.6 μg/L (normal range: 15.00–150.00 μg/L), a serum iron level of 7.45 μmol/L (normal range: 6.6–26 μmol/L), a total iron‐binding capacity (TIBC) of 48.45 μmol/L (normal range: 40.8–76.6 μmol/L), and a transferrin saturation (TSAT) of 15.4%. Furthermore, intact parathyroid hormone (iPTH) level was markedly elevated at 882 pg/mL (normal range: 15–65 pg/mL). Screening tests for autoantibodies, hepatitis viruses, bilirubin abnormalities, Coombs' positivity, antineutrophil cytoplasmic antibodies, and occult gastrointestinal bleeding were all negative. Details of the laboratory findings are presented in Table 1. The electrocardiogram (ECG) revealed left ventricular hypertrophy and ischemic ST‐T changes. No significant abnormalities were observed on chest computed tomography (CT). Abdominal CT demonstrated atrophy of both kidneys, abnormal shape and density of the transplanted kidneys, and a small amount of ascites in the abdominal cavity.

**TABLE 1:** Baseline laboratory parameters at Roxadustat initiation.

Parameter	Recorded value	Reference range (unit)
White blood cell count	4.25	3.50–9.50 (×10^9^/L)
Red blood cell count	3.58	3.80–5.10 (×10^12^/L)
Hemoglobin	91.00	115–150 (g/L)
Platelet count	309.00	125–350 (×10^9^/L)
Calcium	2.16	2.15–2.57 (mmol/L)
Serum phosphate	1.67	1.05–1.70 (mmol/L)
Estimated glomerular filtration rate	6.00	90–110 (mL/min/1.73 m^2^)
Blood urea nitrogen	22.29	1.70–8.30 (mmol/L)
Serum creatinine	811.00	45–84 (μmol/L)
Ferritin	112.60	15–150 (μg/L)
Serum iron	7.45	6.60–26 (μmol/L)
Total iron‐binding capacity	48.45	40.80–76.60 (μmol/L)
Transferrin saturation	15.40	14–50 (%)
High‐density lipoprotein	0.87	1.04–1.55 (mmol/L)
Triglyceride	2.38	< 1.7 (mmol/L)
Cholesterol	5.54	< 5.18 (mmol/L)
Low‐density lipoprotein	3.44	< 3.37 (mmol/L)
25‐OH vitamin D	10.00	> 20 (ng/mL)
Intact parathyroid hormone level	882.00	16–65 (pg/mL)
Sucrose hemolysis test	Negative	Negative
Direct Coombs' test	Negative	Negative
EPO concentration (mIU/mL)	Not detected	—
Anti‐EPO antibodies	Not detected	—

*Note:* Units are indicated in column headers; data reflect the time of Roxadustat initiation.

Based on the above findings, the final diagnoses included chronic kidney disease stage 5 on maintenance hemodialysis, renal anemia, nephrogenic secondary hyperparathyroidism, grade 3 hypertension in a very high‐risk category, and ischemic heart disease. A comprehensive evaluation was performed to investigate the etiology of the persistent anemia. The patient exhibited no evidence of gastrointestinal bleeding, hemolysis, systemic inflammation, folic acid or iron deficiency at baseline, or inadequate dialysis efficacy (Kt/V > 1.5). Subcutaneous ESAs were initiated at a dose of 4000 IU twice weekly (BIW), calculated based on a dry weight of 42 kg. Paricalcitol was concurrently prescribed to manage secondary hyperparathyroidism. The baseline hemoglobin (Hb) level was 91 g/L. However, the Hb level declined to 81 g/L after 1 month and remained suboptimal (89 g/L) at 2 months, indicating an inadequate hematologic response. During this period, the patient continued to experience anemia‐related symptoms, including tachycardia, fatigue, and exertional dyspnea.

**FIGURE 1:**
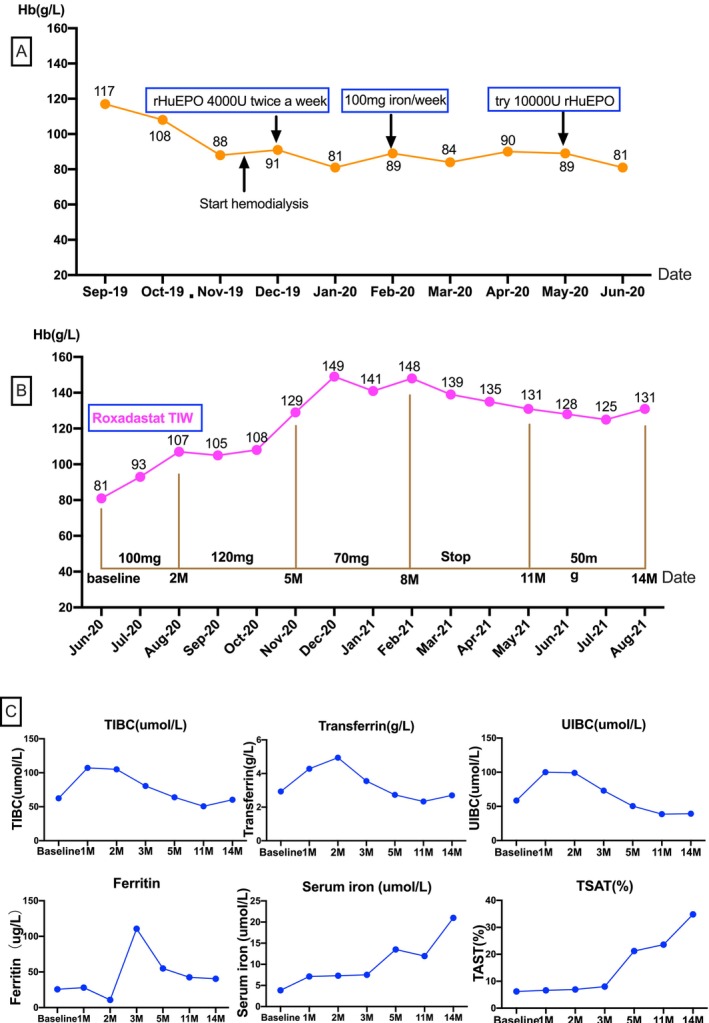
Changes in hemoglobin (Hb) levels during ESA and Roxadustat treatment (top), and changes in iron metabolism markers after Roxadustat initiation (bottom). ESA, erythropoiesis‐stimulating agent; TIBC, total iron‐binding capacity; TSAT: Transferrin saturation; UIBC, unsaturated iron‐binding capacity.

Further investigation of potential causes for hyporesponsiveness revealed no significant inflammation, advanced hyperparathyroidism, or occult blood loss. However, after 2 months of hemodialysis, the patient developed iron deficiency, as indicated by a normal SF level of 25.7 μg/L, decreased serum iron of 3.85 μmol/L, and TSAT of 6.2%. Intravenous iron (1000 mg) was administered, but the patient's anemia did not improve (Figure 1A). A higher dose of recombinant human EPO (rHuEPO, 10,000 IU/week) was then attempted but discontinued due to intolerable, severe headaches.

Given the persistence of anemia, inadequate response to ESA, and poor treatment tolerance, oral Roxadustat therapy was initiated with informed consent from the patient's guardians. Based on a dry weight of 42 kg, the starting dose was 100 mg three times per week (TIW), administered post‐dialysis. Prior to the initiation of Roxadustat, the patient's hemoglobin (Hb) level was 81 g/L (Figure 1B). Over the following 2 months, her Hb increased gradually to 93 g/L in the first month and 107 g/L in the second. Concurrently, she reported marked relief from symptoms of weakness, fatigue, and exertional dyspnea. The Roxadustat dose was subsequently increased to 120 mg three times weekly (TIW) over the next 3 months. During this period, her Hb rose rapidly, exceeding 140 g/L, and anemia‐related symptoms continued to improve. As shown in Figure 1B, the dose was titrated according to Hb levels, and a maintenance dose of 50 mg TIW was sufficient to sustain hemoglobin within the target range. With anemia correction, the patient's cardiac function improved, and her blood pressure became more stable than before.

In addition to improvements in hemoglobin levels, iron metabolism parameters also demonstrated significant enhancement. As shown in Figure 1C, levels of TIBC, transferrin, unsaturated iron‐binding capacity (UIBC), and ferritin increased markedly during the first 3 months of treatment, before stabilizing within normal ranges. Serum iron and TSAT also showed a steady upward trend, ultimately reaching optimal levels.

Throughout the treatment and follow‐up period, no serious adverse events such as peripheral edema, vomiting, myalgia, rash, elevated blood pressure, increased alanine aminotransferase levels, metabolic acidosis, or nausea were observed. Only mild hyperkalemia was occasionally noted, which had already been present prior to the initiation of Roxadustat therapy. The treatment of Roxadustat significantly improved the patient's quality of life. The patient continued to take oral Roxadustat at a dose of 50 mg per dose (TIW) for a period, and after 3 months of cessation, her Hb stabilized at approximately 100–110 g/L.

## 3 Discussion and Conclusion

### 3.1 Efficacy

Anemia is common in hemodialysis patients due to impaired renal EPO production and iron dysregulation [1, 2]. While ESAs have improved outcomes in ESRD, pediatric patients often exhibit poor responses due to higher inflammation, blood loss, and EPO requirements, making ESA resistance or intolerance easily overlooked in this group [6].

We report an adolescent patient on maintenance hemodialysis patient with anemia unresponsive to standard ESA therapy. Common contributors to ESA hyporesponsiveness include iron deficiency, malnutrition, vitamin deficits, inadequate dialysis, inflammation, oxidative stress, and secondary hyperparathyroidism.

In our patient, iron deficiency was confirmed through iron metabolism tests, and intravenous iron (1000 mg) was administered, but the patient's anemia did not improve. Secondary hyperparathyroidism (SHPT) is also recognized as a contributor to hyporesponsiveness. Elevated parathyroid hormone can disrupt erythropoiesis by causing marrow fibrosis, shortening red cell lifespan, and affecting iron metabolism. This patient had a markedly high PTH level (882 pg/mL) and was treated with paricalcitol, but anemia persisted. Due to ESA intolerance from severe headaches, Roxadustat was started, resulting in marked hemoglobin improvement.

Clinical trials in adult hemodialysis populations have consistently demonstrated the efficacy and tolerability of Roxadustat in managing ESA‐hyporesponsive anemia [7]. However, data on its use in pediatric populations remain limited. Yang et al. (2024) reported the successful use of Roxadustat in an infant (80 days old; weight 4.2 kg): hemoglobin increased from 74 to 111 g/L within 8 weeks as the dose was uptitrated from 10 mg TIW (≈2.4 mg/kg) to 20 mg TIW (≈4.8 mg/kg), thereafter remaining around 120 g/L for > 2 years, suggesting promising potential in younger patients [8]. By contrast, our adolescent maintained approximately 100–110 g/L on 50 mg TIW over 5 years of follow‐up. Further investigations are now underway to evaluate Roxadustat's role in pediatric CKD. In addition, two pediatric trials further inform dosing and follow‐up. A single‐center Chinese Phase 3 study (NCT04925011) in children/adolescents with CKD stages 3–5 (dialysis and non‐dialysis) uses body‐weight and dialysis‐status–stratified starting doses (ranges reported as 20–100 mg vs. 30–120 mg TIW) with the primary efficacy window at Weeks 16–24 (proportion with mean Hb ≥ 11.0 g/dL); eligibility requires ferritin > 50 ng/mL and TSAT > 10%. As of now, no study results have been posted or published [9]. A global multicenter Phase 3 study (NCT05970172) treats participants TIW for up to 52 weeks with a fixed dose for the first 4 weeks followed by titration to maintain Hb just below normal, and requires ferritin > 100 ng/mL or TSAT > 20%; results are pending [10]. Taken together, the limited pediatric evidence—supported by our case and early reports, together with the design of ongoing trials—suggests that Roxadustat may be a viable alternative for ESA‐hyporesponsive or ESA‐intolerant pediatric CKD, pending confirmation from prospective studies.

### 3.2 Iron Metabolism Effects

Inflammation from uremia or dialysis, marked by high IL‐6 and C‐reactive protein (CRP), contributes to ESA hyporesponsiveness; Roxadustat remains effective even under inflammatory conditions [11]. Moreover, Roxadustat directly increases transferrin levels through HIF pathway activation, as the transferrin gene contains two HIF‐responsive elements within its 5′ enhancer region [12]. In addition, by suppressing hepcidin, Roxadustat further facilitates dietary iron absorption and mobilization, although the precise mechanism underlying its hepcidin‐lowering effect remains to be fully elucidated [13].

In our case, transferrin, UIBC, TIBC, and ferritin levels rose initially and then stabilized, while serum iron and TSAT showed a sustained increase. These changes suggest Roxadustat enhances iron absorption, transport, and utilization, supporting erythropoiesis. Preclinical data also suggest that Roxadustat may reduce fibrosis, improve mitochondrial function, and normalize angiogenesis [14]. These aspects were not evaluated in our case but warrant consideration in future studies.

### 3.3 Safety Considerations

A key concern in the use of Roxadustat, particularly in children and adolescents, is its safety profile. Roxadustat demonstrated good tolerability in phase 3 trials conducted among Chinese and Japanese patients with either dialysis‐dependent or non‐dialysis chronic kidney disease [15, 16, 17, 18]. Common treatment‐emergent adverse events such as nasopharyngitis, back pain, diarrhea, vomiting, and hypertension were not observed during treatment in our case. It has been hypothesized that HIF stabilization may elevate the risk of neoplasia and diabetic retinopathy by promoting overexpression of vascular endothelial growth factor (VEGF). However, evidence from clinical trials indicates that VEGF levels remain largely unchanged across various doses of Roxadustat [19]. In addition, Roxadustat has demonstrated efficacy in managing anemia among cancer patients without accelerating tumor growth [20]. While positive outcomes have been reported, observational signals have raised concerns about possible associations with pulmonary hypertension [21], vascular calcification [22], and infectious complications [23]. Consequently, careful management of the extent and duration of HIF activation, as well as thoughtful dosing and scheduling of Roxadustat, is necessary to avoid potential side effects.

## 4 Conclusion

This case highlights the effective use of Roxadustat in a 13‐year‐old hemodialysis patient with ESA hyporesponsiveness and intolerance. However, several limitations should be considered. First, due to financial constraints, serum EPO levels and anti‐EPO antibody titers were not assessed. Second, the improvement observed in iron metabolism parameters cannot be exclusively attributed to Roxadustat, as early‐stage iron supplementation may have played a contributory role. Third, although no major adverse events were observed over 5 years of follow‐up (the patient is now 18 years old), longer‐term surveillance remains necessary, particularly for adolescent populations. In conclusion, Roxadustat may be a viable alternative for pediatric patients unresponsive or intolerant to ESA therapy, but ongoing monitoring is essential. Future prospective studies are warranted to further validate the therapeutic efficacy and long‐term safety profile of Roxadustat.

## Author Contributions

**Meng Zhang:** conceptualization, investigation, validation. **Yanchao Guo:** conceptualization, investigation, validation. **Yan Zhu:** data curation, project administration. **Juan Yang:** data curation, formal analysis, writing – original draft, writing – review and editing.

## Ethics Statement

The Ethics Committee of the Tongji Hospital, Tongji Medical School of Huazhong University of Science and Technology approved the off‐label use of Roxadustat (TJ‐IRB20211275).

## Consent

Written informed consent was obtained from the patient's parents. Written informed consent for publication of this case report and any accompanying images was obtained from the patient's legal guardian.

## Conflicts of Interest

The authors declare no conflicts of interest.

## Data Availability

The datasets generated during and/or analyzed during the current study are available from the corresponding author on reasonable request.
